# Impacts of Cigarette Smoking on the Tumor Immune Microenvironment in Esophageal Squamous Cell Carcinoma

**DOI:** 10.7150/jca.65400

**Published:** 2022-01-01

**Authors:** Geng Wang, Chuqing Pan, Kexin Cao, Jingbing Zhang, Hui Geng, Kusheng Wu, Jing Wen, Caixia Liu

**Affiliations:** 1 Department of Preventive Medicine, Shantou University Medical College, Shantou, Guangdong, China.; 2 Department of Thoracic Surgery, Cancer Hospital of Shantou University Medical College, Shantou, Guangdong, China.; 3 State Key Laboratory of Oncology in South China, Collaborative Innovation Center for Cancer Medicine, Sun Yat-sen University Cancer Center, Guangzhou, China.; 4 Guangdong Esophageal Cancer Research Institute, Guangzhou, China.

**Keywords:** Cigarette smoking, Esophageal squamous cell carcinoma, Immune infiltration, PD-L1, Tumor microenvironment

## Abstract

**Objective:** Cigarette smoking is a carcinogenic factor for esophageal cancer and evidence also indicates its effects on tumor microenvironment in patients with esophageal squamous cell carcinoma (ESCC).

**Materials and Methods:** In our study, we demonstrated nine immune infiltrating cells and markers in non-smokers and smokers of 189 non-drinking ESCC patients with multiplex fluorescent immunohistochemistry (mflHC) staining and multispectral imaging. The impacts of cigarette smoking on tumor microenvironment and patient prognosis were also analyzed.

**Results:** Among 189 ESCC patients of non-drinker, 86 patients was current smokers, while 34 males and 59 females were non-smokers and 10 former-smokers. Among 34 male non-smokers and 83 smokers, distinct immune infiltrating cells, with increased DCs in stromal regions (*P*=0.033), elevated infiltration of Treg cells in intraepithelial regions (*P*=0.010) and reduced activate cytotoxic T lymphocytes (aCTLs) in both intraepithelial (*P*=0.021) and stromal regions (*P*=0.017), were observed in tumor specimens of smoking males, implying an immune suppressed response during cigarette smoke exposure. For smoking characters, the level of stromal tumor-associated macrophages (TAMs) infiltration was correlated with smoking year after age adjusted (*r_s_*=0.352, *P*=0.002). Though cigarette smoking did not alter the expression of programmed death ligand 1 (PD-L1) in epithelial cells or TAMs in tumor specimens, higher expression of PD-L1 predicted a worse survival in non-smokers but not smokers.

**Conclusions:** Our findings indicated smoking may impair T cell-mediated immune response and supported the possible impacts of cigarette smoking in PD-L1 related research and therapy of ESCC.

## Introduction

Esophageal cancer, which mainly consists of two histopathological subtypes: esophageal adenocarcinoma (EAC) and esophageal squamous cell carcinoma (ESCC), causes almost 0.5 million death globally according to the GLOBOCAN statistics, with a poor 5-year survival rate approximately 20%^1^. China accounts for more than 50% of the global morbidity and about 90% of cases was ESCC^2^. Tobacco smoking strongly increases the risk of ESCC. A meta-analysis including 41 ESCC studies indicated that current smokers have a 4-fold higher risk for ESCC compared to nonsmoker, while quitting smoking suggested a significantly decreased likelihood of getting ESCC with cessation duration dependently^3^. In China, the smoking prevalence among people 15 years old and above from 2003 to 2013 was 24.5% and most of the smokers were males^4^. As studies reported, about 50% of ESCC cases were attributable to smoking^5^, ^6^.

Cigarette smoke contains thousands of chemicals, including more than 60 carcinogens, such as polycyclic aromatic hydrocarbons (PAHs), N-nitrosamine, aromatic amines and so on 7. Carcinogens in cigarette smoke cause cancers *via* various mechanisms. Most of cigarette smoke carcinogens were metabolically activated and covalently bind to DNA, forming DNA adducts and resulting somatic mutations. Some receptors (e.g., AhR and nAChR ) directly mediate pathways which regulated cell growth, apoptosis, angiogenesis, and transformation, while cigarette smoke also led to aberrant methylation or some other epigenetic change during carcinogenesis^8^. However, cigarette smoke exposure not only dysregulated cell per se but also modified host microenvironment to favor tumorigenesis and invasion of cancer cells, which was always overlooked during *in vitro* research. By either cytotoxic activity impairment or cytokine releasing of tumor inflammatory cells, cigarette smoke impacts both innate and adaptive immunity in lung, breast, and colorectal cancer^9^-^12^.

The biology of a tumor was accepted as the individual specialized cell types construction, as well as “tumor microenvironment” during the process of multistep tumorigenesis^13^. ESCC was not an exception. Esophageal intraepithelial neoplasia, a precancerous lesion of ESCC which was significantly increased the risk for esophageal cancer, was thought to a chronic inflammation^14^.Studies based on single cell RNA sequencing presented an immune suppressive landscape in ESCC, including exhausted T and NK cells, regulatory T cells (Tregs), alternatively activated macrophages and tolerogenic dendritic cells 15. With chemical carcinogen treatment, a mice model mimicking human ESCC development also was found distinguishing fibroblast and CD T cell clusters, suggesting a turndown of adaptive anticancer immune response during tumorigenesis^16^. Data of a phase II clinical trial of PD-L1 inhibitor has suggested the durable antitumor for PD-L1 positive patient, implying the immune escape mechanism among tumor occurrence and development^17^. Though many studies of lung cancer have revealed the different effect of cigarette smoker and non-smoker about tumor microenvironment, how cigarette smoke alters ESCC immune infiltrating is largely unknown. In our previous study^18^, heterogeneous immune population infiltrating intraepithelial and stromal region, which was associated with overall survival, was observed. In this study, we further analyzed the effect of cigarette smoking on the immune cell infiltration of ESCC tissue samples.

## Materials and Methods

### Study population

A total of 189 smoking or non-smoking patients of ESCC pathologically staged Ib-IIIc(the 8^th^ edition of AJCC), were recruited and underwent complete esophagectomy at the Department of Thoracic Surgery in Sun Yat-sen University Cancer Center from Sep. 2002 to Jul. 2012. The criteria for the inclusion of ESCC patients were (1) resectable esophageal squamous cell carcinoma, (2) no distant metastases of the tumor. And the criteria for exclusion were (1) either alcohol consumption or long-term medication, (2) serious complications or other malignant disease, (3) chemotherapy and/or radiotherapy before the operation. After the surgery, the patient was followed more than 5 year with a frequency for every month during the first 3 months, every 3 months during the following 9 months, every 6 months during the second year and every year after that until recurrence, death or 10 year. Overall survival (OS) was referred to the time from surgery to death or the last follow-up from any cause and disease-free survival (DFS) to the date with any signs or symptoms of ESCC. Tissue microarrays were performed with formalin-fixed paraffin-embedded (FFPE) blocks of archived tumor (n=189) or normal (n=29) tissues from ESCC patients. Two 1-mm cores for the representative areas of specimens were punched and arrayed on a recipient paraffin block, as described previously. The study were approved by the Ethics Committee of Sun Yat-sen University Cancer Center (GZR2020-275). Overview flowchart of this study was present in the Figure S1.

### Multiplex fluorescent immunohistochemistry (mflHC) staining and multispectral imaging

The PANO Multiplex IHC kit (Panovue, Beijing, China) was used to examine specific markers of immune cells, including CD11c (Abcam), CD45RO (Cell Signaling), CD68 (ZSGB-Bio), panCK (Cell Signaling), and PD-L1 (Cell Signaling) in panel A, and CD4 (biolynx), CD8A (Cell Signaling), CD56 (Cell Signaling), FoxP3 (Biolegend), and granzyme B (Abcam) in panel B in two 4-µm sections from tissue microarrays blocks. Primary antibodies were applied, followed by horseradish peroxidase conjugated secondary antibody incubation. Then the slides were microwave heat-treated after tyramide signal amplification operation. Lastly, DAPI (Sigma-Aldrich) were subjected to stain the nuclei after all antigens had been labeled.

A Vectra Multispectral Imaging System (PerkinElmer) was used for getting images. One image per core was captured at 200x magnification and each multispectral image cube was performed by combining images obtained every 10 nm of emission light spectrum across the range of emission filter cube. Five filter cubes, including DAPI (440-680 nm), FITC (520-680 nm), CY3 (570-690 nm), CY5 (670-720 nm), and Texas Red (580-700 nm), were used for each image capturing.

### Image analysis

InForm Cell Analysis software (PerkinElmer) was used to analyze images of all available cores. With antigen staining for each fluorophore, a library was made and multispectral images were unmixed with color-based identification. Based on panCK(pan Cytokeratin, characteristic of epithelial cell ) staining, intraepithelial (IE) and stromal (ST) regions was identified by an algorithm. Cells were phenotype as normal or tumoral epithelial cells (ECs) (panCK^+^), Tumor-associated macrophages (TAMs, CD68^+^), Dendritic cells (DCs, CD11c^+^), memory T cells (Tmems, CD45RO^+^), T helper cells (Ths, CD4^+^FoxP3^-^CD8A^-^), regulatory T cells (Tregs, CD4^+^FoxP3^+^CD8A^-^), Cytotoxic T lymphocytes (CTLs, CD8A^+^CD4^-^CD56^-^), granzyme B^+^ activate cytotoxic T lymphocytes (aCTLs, granzyme B^+^CD8A^+^CD4^-^CD56^-^), and natural killer cells (NKs, CD56^+^). In addition, programmed death ligand 1 (PD-L1^+^) were marked and intensity of the markers above in all compartments was recorded. The representative images were shown in Figure S2.

### Smoking assessment

Smoking characteristics of patients were record by self-report when initial diagnosis. Cigarette smokers were defined as patients smoked at least once per day for more than one year. If patients reported cigarette smoking at initial diagnosis or quitted within 1 year of consultation, they were considered as current smokers. The variables about smoking exposure included smoking status (never and current smokers), duration of smoking (smoking year), pack-years of smoking. Pack-years of smoking is calculated by multiplying pack number of cigarettes per day by smoking duration in years.

### Statistical analysis

The ratio of each immune cell to total cell was analyzed in epithelial and nonepithelial regions which were segmented based on epithelial cell (EC) staining in normal specimens, as well as the tumor tissues. Statistical analysis was performed with SPSS software 26.0 (IBM Corp, NY, US). Categorical variables were described with frequencies and proportions and continuous variables with medians and interquartile ranges (IQRs). Between groups comparison, the *chi*-squared (or *Fisher* exact test) and *Mann-Whitney* test were used categorical and continuous variables, respectively. For correlation between immune infiltration, age and cigarette characteristics, Spearman's rank correlation test or partial correlation was performed. All tests were two-sided, and *P*≤0.05 was defined as statistically significance. Survival probability difference was assessed with log-rank test. Multivariable Cox regression analysis were employed to evaluate the factors that impact on survival prognosis between smoker and non-smokers, adjusting with age, tumor size, T stage, N stage, G stage, smoking status in each model.

## Results

### Immune populations infiltrate in normal esophageal epithelial cells between smoker and non-smokers

**Figure 1:**
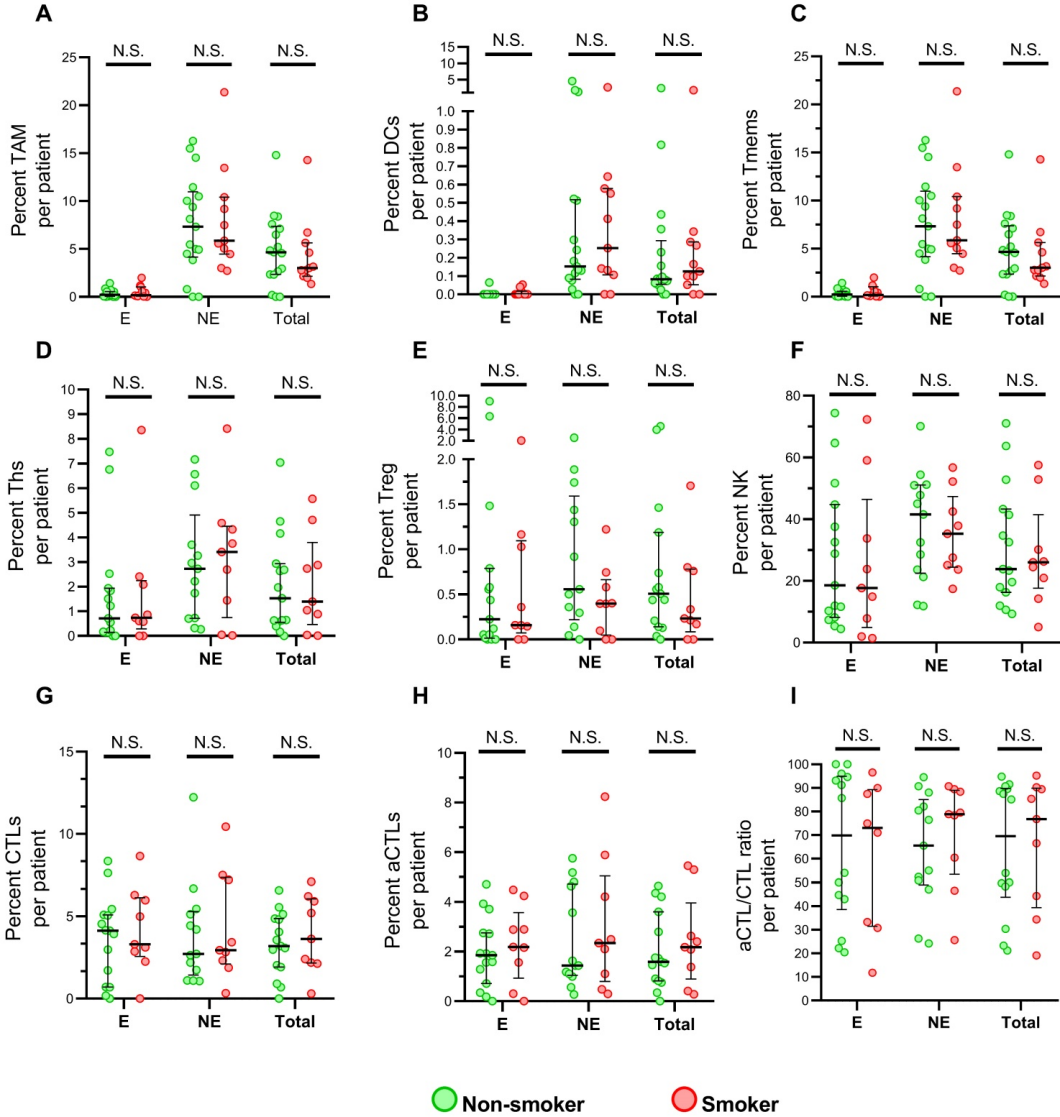
** Immune populations infiltrating in normal esophageal epithelial cells between smoker and non-smokers.** (A-I) Grouped comparisons of immune subpopulations in epithelial and nonepithelial regions per patient for TAM (A), DCs (B), Tmems (C), Ths (D), Treg (E), NK (F), CTLs (G), aCTLs (H) and aCTL/CTL ratio (I) between smokers(n=12) and non-smokers(n=17). The data was presented with median and interquartile range. E, epithelial region. NE, nonepithelial regions. N.S. no significance.

The immune infiltrates from normal were analyzed to explore the impact of cigarette chronic exposure. In total, twenty-nine normal tissue were acquired for mflHC staining and eight phenotype cells, tumor-associated macrophages (TAMs), dendritic cells (DCs), memory T cells (Tmems), T helper cells (Ths), regulatory T cells (Tregs), cytotoxic T lymphocytes (CTLs), granzyme B+ activate cytotoxic T lymphocytes (aCTLs), and natural killer cells (NKs) were compared. The clinical characteristics of these patients were shown in supplementary Table S1. Generally, NK cells have a significantly higher immune infiltration than the other phenotype cell, while DCs and Tregs presented the lowest infiltration (Figure 1). Additionally, immune infiltrated cells were more abundant in epithelial than nonepithelial regions, expect for CTLs and aCTLs. CTL have the similar activated distribution between epithelial, nonepithelial regions, according to the ratio of aCTLs to CTLs. No significant difference of immune cell infiltration was found between smokers and non-smokers among either epithelial, nonepithelial regions or total uninvolved tissue core, implying no obvious influence of cigarette smoking on immune in uninvolved esophageal cell.

### Distinct immune infiltrating in tumor specimens of ESCC

Among 189 ESCC patients of non-drinker, 93 patients (49.2%) of them did not smoking, including 34 males and 59 females, while10 of them (5.3%) have quit smoking and 86 patients (45.5%) were current smoker. Since only 3 females (3.5%) were current smoker, we only focus on 34 male non-smokers and 83 current smokers for further analyzing the impact of cigarette smoking on tumor infiltrating, excluding the effect of gender and smoking cessation. The prognostic associations of clinical characteristics were analyzed in subgroup of smoking status. With multivariate Cox regression analysis (Table 1), older non-smoking patients (>58 yrs, HR=3.36, 95%CI: 1.05, 10.69) and positive lymph node smokers (N1-3, HR=3.34, 95%CI: 1.63, 6.83) have a shorter overall survival time. And smoking status was not a predictor for survival.

**Figure 2:**
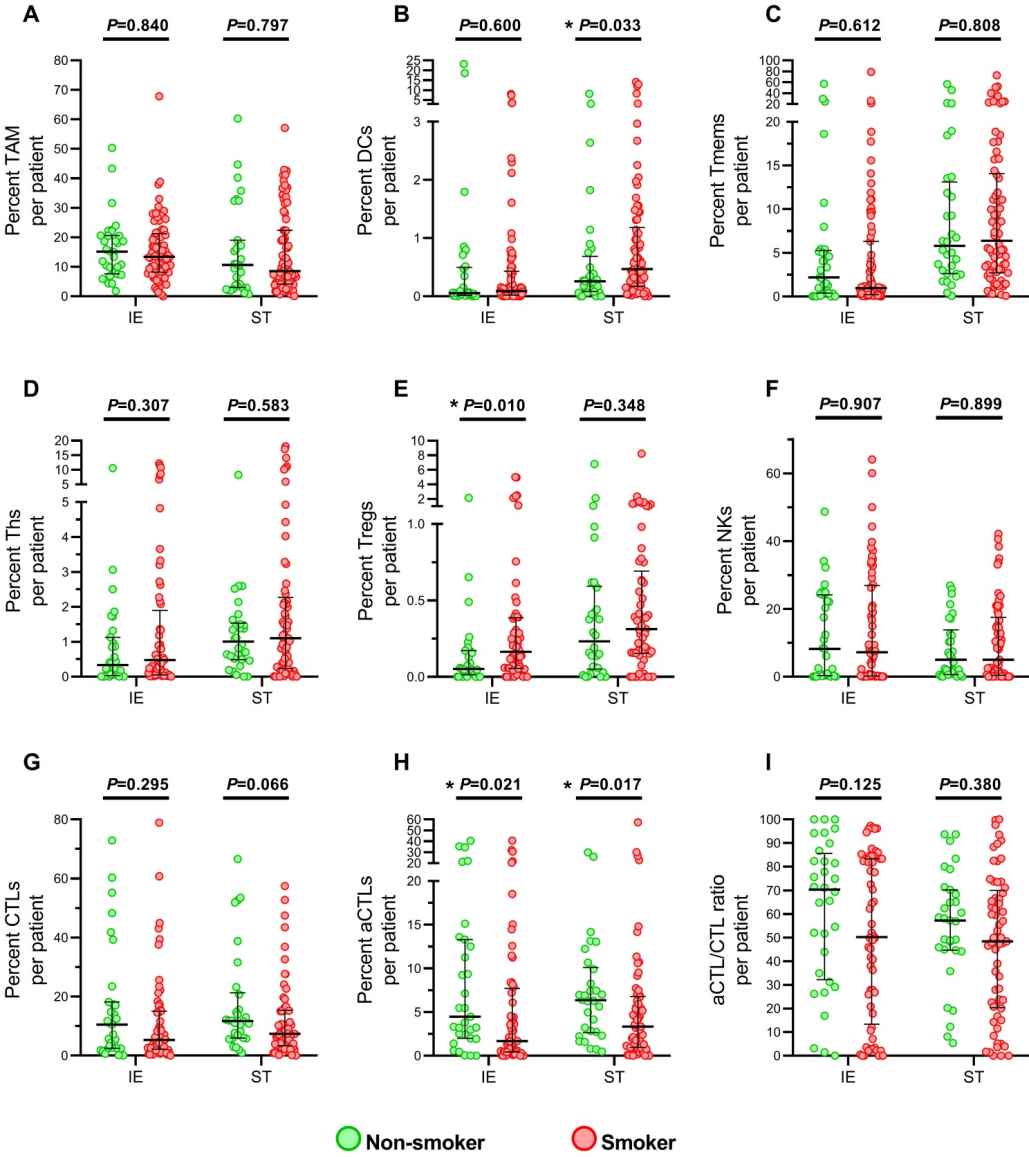
** Immune infiltrating cells in tumor specimens between smoking and non-smoking ESCC patients.** (A-I) Grouped comparisons of immune subpopulations in intraepithelial and stromal regions per patient for TAM (A), DCs (B), Tmems (C), Ths (D), Treg (E), NK (F), CTLs (G), aCTLs (H) and aCTL/CTL ratio (I) between smokers(n=83) and non-smokers(n=34). The data was presented with median and interquartile range. IE, intraepithelial region. ST, stromal region. * *P*< 0.05.

No difference between non-smokers and current smokers was found in age, tumor size, T stage, N stage, TNM stage and differentiation grading (Table S2). Then, the immune infiltration level was compared (Figure 2). The results showed that smokers presented an elevated level of Tregs (Figure 2E) and decreased aCTLs (Figure 2H) in intraepithelial regions, while significant increased DCs (Figure 2B) and lower level of aCTLs (Figure 2H) were found in stromal regions under cigarette smoke exposure. And no significant difference between smokers and non-smokers was observed in TAMs, Tmems, Ths, NKs, CTLs in both regions and DCs in intraepithelial regions, Tregs in stromal regions. Though aCTL/CTL ratio in intraepithelial and stromal regions (Figure 2I) were downregulated in smokers, no statistical significance was reached. And the change in level of aCTLs in intraepithelial and stromal regions, DCs in stromal regions and Tregs intraepithelial regions did not predicted either better or worse survival (Figure S3).

### Association between immune infiltration, age, and smoking characteristics

**Figure 3:**
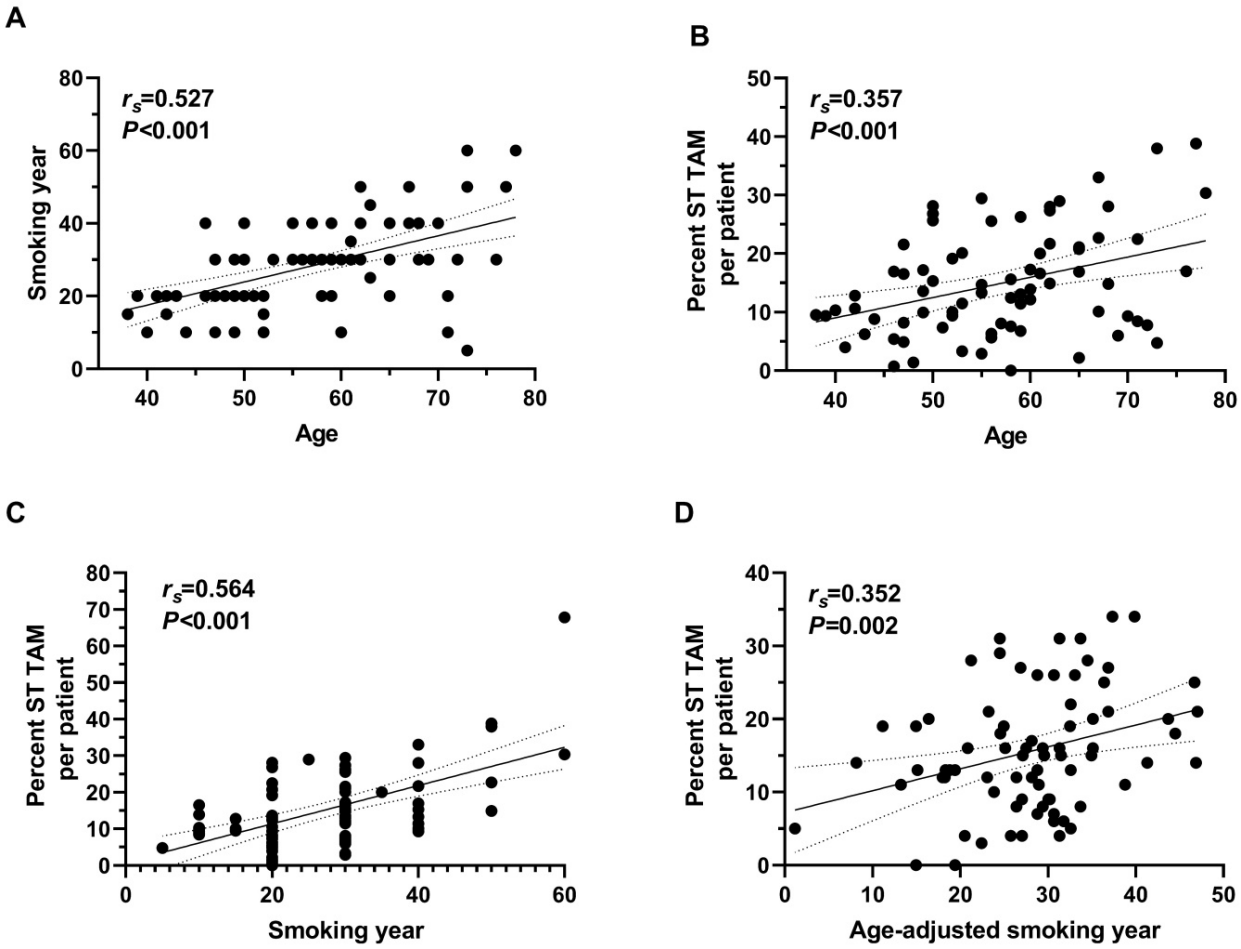
** Correlation between age, smoking year and TAM infiltrating in stromal regions of tumor tissue in smoking ESCC patients.** (A) Correlation between age at diagnosis and smoking year (n=83). (B) Correlation between age at diagnosis and TAM in stromal regions (n=77). (C) Correlation between smoking year at diagnosis and TAM in stromal regions (n=78). (D) Correlation between smoking year and TAM in stromal regions after age adjusted (n=76). ST, stromal region. *r_s_*, Spearman correlation coefficient.

Since younger nonsmoking males have better survival, we also analyzed the correlation between immune infiltration, age, and smoking characteristics. Intriguingly, level of Tregs infiltration in intraepithelial regions was positively correlated with age (*r_s_*=0.39, *P*=0.032) and NKs in intraepithelial negatively with age in male non-smokers (*r_s_*=-0.37, *P*=0.043) (Table S3). In smokers, TAM infiltration in intraepithelial and stromal regions (*r_s_*=0.33, *P*=0.003 and *r_s_*=0.36, *P*=0.001, respectively), as well as NKs in stromal regions (*r_s_*=0.39, *P*=0.002) were correlated with age (Table S3, S4). As for immune infiltration and smoking characteristics, more pack-years of cigarette smokers took, more TAM infiltrated in stromal regions (*r_s_*=0.25, *P*=0.029), while TAM infiltrated more in intraepithelial and stromal regions (*r_s_*=0.26, *P*=0.020 and *r_s_*=0.47, *P*<0.001) in smokers exposed for more years (Table S4). Since older smokers always have longer smoking duration, we analyzed the association between immune infiltrated level and characteristics after age adjusted (Table S4). And only positive correlation between TAM infiltration in stromal infiltration and smoking year was found, with an approximate correlation coefficient with that between age and TAM infiltration in stromal regions (*r_s_*=0.35, *P*=0.002) (Figure 3).

### PD-L1 expression in immune cell and different impacts on survival between smoking and non-smoking ESCC patients

**Figure 4:**
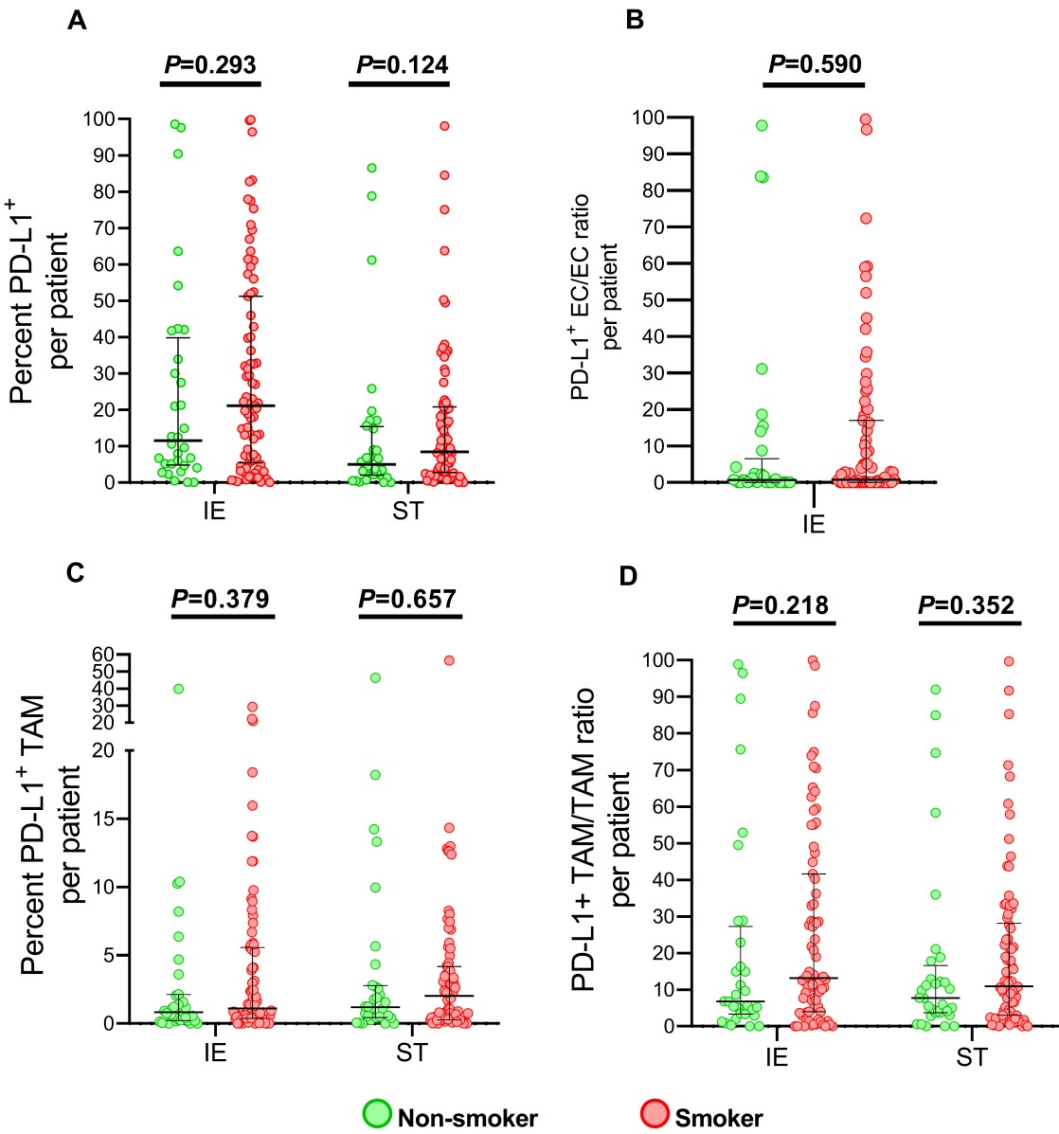
** PD-L1 expression infiltrating immune cells in tumor specimens between smoking and non-smoking ESCC patients.** Grouped comparisons of all cells expressing PD-L1 (A), PD-L1^+^ EC/EC ratio (B), TAM expressing PD-L1 (C) and PD-L1^+^ TAM/TAM ratio (D) in intraepithelial and stromal regions per patient between smokers(n=77) and non-smokers(n=32). The data was presented with median and interquartile range. IE, intraepithelial region. ST, stromal region.

Since PD-L1 expression was induced by cigarette smoke in lung epithelial cell and predicted immune checkpoint inhibitor efficacy in metastatic lung cancer patients^19^, ^20^, we also analyzed the level of PD-L1 in TAMs and ECs, which were the major components expressing in tumor microenvironment. As presented in Figure 4, no significant elevating PD-L1 expression of infiltrated TAMs, as well as ECs, was observed among smokers, though percent of PD-L^+^ cells in intraepithelial regions showed slightly increased smoking patients.

**Figure 5:**
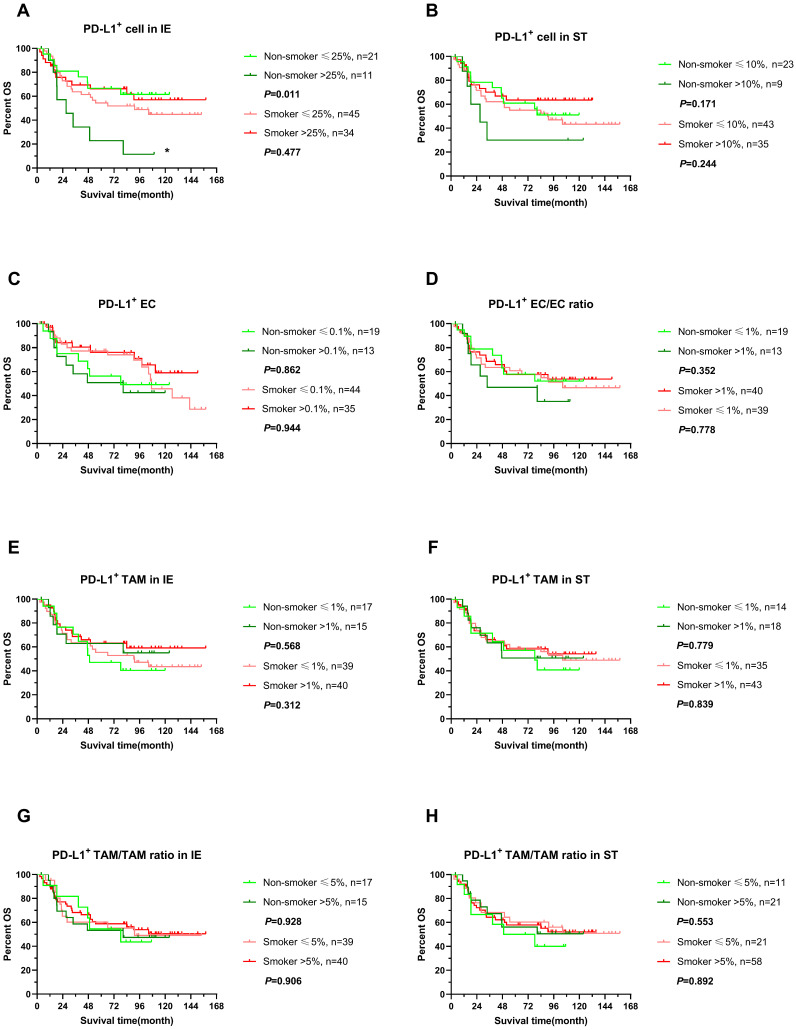
** OS of stratified immune cell infiltration in tumor specimens between smoking and non-smoking ESCC patients.** (A-H) OS comparisons of immune subpopulations in intraepithelial and stromal regions per patient for PD-L1^+^ cells (A, B), PD-L1^+^ ECs (C, D), PD-L1^+^ TAM (E, F), PD-L1^+^ TAM/TAM ratio (G, H) in smokers(red line) and non-smokers(green line), IE, intraepithelial region. ST, stromal region. * *P*< 0.05.

**Figure 6:**
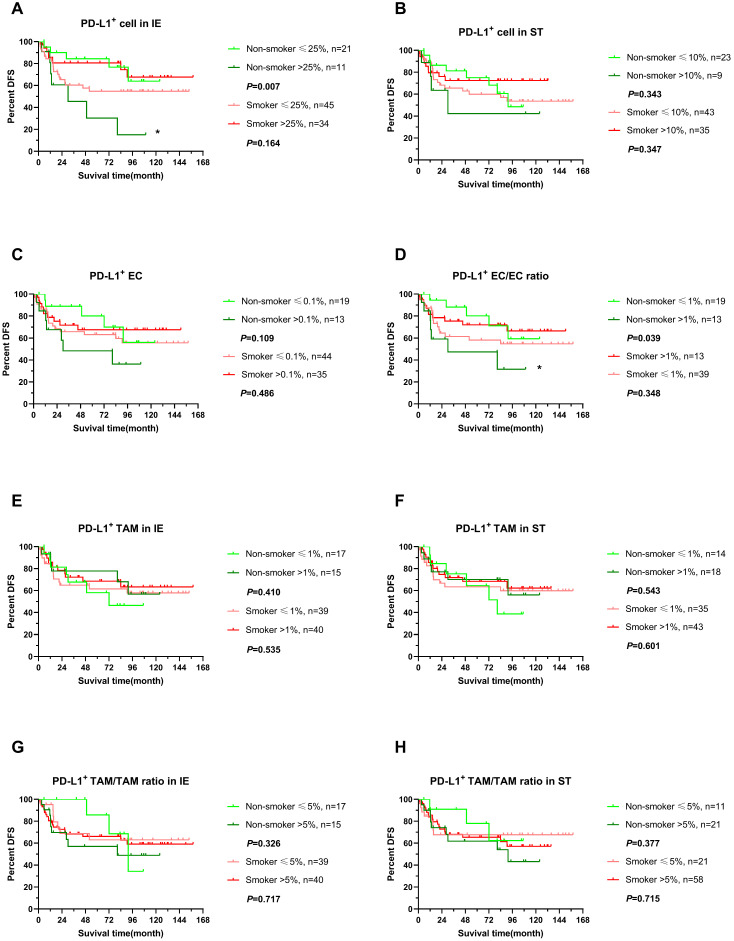
** DFS of stratified immune cell infiltration in tumor specimens between smoking and non-smoking ESCC patients.** (A-H) DFS comparisons of immune subpopulations in intraepithelial and stromal regions per patient for PD-L1^+^ cells (A, B), PD-L1^+^ ECs (C, D), PD-L1^+^ TAM (E, F), PD-L1^+^ TAM/TAM ratio (G, H) in smokers(red line) and non-smokers(green line), IE, intraepithelial region. ST, stromal region. * *P*< 0.05.

We further explored the impact of PD-L expression on survival of ESCC in non-smokers and smokers by stratifying into high or low level in infiltrating cells, which was determined by cutoff with X-tile with adjusted. Interestingly, higher expression of PD-L1 in intraepithelial regions were associated with poor clinical outcomes, OS and DFS included, in non-smokers (*P*<0.05), while PD-L1 expressing level did not influence survival in smokers (Figure 5A, 6A). In addition, PD-L1+ EC/EC ratio decreased also predicted a prolonged DFS in non-smokers but not for smokers (Figure 6D) (*P*=0.039). There was no significant correlation between PD-L1 expression level of TAM, PD-L1+ TAM/TAM ratios in intraepithelial and stomal regions in either non-smokers or smokers.

## Discussion

Inflammatory microenvironment plays an important role in cancer development, including esophageal cancer, while tumor inflammatory cells promote carcinogenesis and tumor invasion by releasing supplying cytokine/growth factors and extracellular matrix-modifying enzymes. As a common risk factor for cancer and other diseases, cigarette smoking impacts on both innate and adaptive immunity and plays dual roles to accelerate pathogenic immune responses or weaken defensive immunity^21^. Heterogeneous cell populations implied the complicity of the cellular networks in the tumor microenvironment. Traditional methods in identifying immune cell types, such as chromogenic IHC and flow cytometry, suffer from limitations in multicolor staining, visualizing minimally expressed antigens or difficulty in intercellular distance and interactions *in situ*^22^. Here, we used mfIHC, a novel method promising immune biomarker quantification^23^, markedly increased sensitivity, and single-cell resolution, to examine the change of multiple immune cell subpopulations under the chronic cigarette smoke exposure. In the study, we found a diverse immune cell infiltration in smokers' tumor specimens and smoking characteristics related cell populations, as well as different prognosis impacts of PD-L1 expression during chronic cigarette smoke exposure.

Tumor immune microenvironment was constructed during multistep carcinogenesis and influence the patient's response to therapy. Infiltrating immune cells was accepted to be generic constituents of tumors and share various duties in regulating innate and adaptive immune functions^13^, ^24^. In addition to high-frequency base mutations, cigarette smoke also switched the immune response to a distinct pattern in shaping the tumor microenvironment^25^. In our study, smoking ESCC patients presented a diverse distribution of infiltrating immune cell populations in tumor tissues. Reduced number of DCs, a subset of professional antigen presenting cells that drive T-cells differentiation, was observed in stromal region of tumor in smoking patients, compared to non-smokers. In immune-edited tumors, DCs suffered from dysfunction antigen procession and presentation; cancer cell can escape from anti-tumor response by releasing of cytokines and other mediators to inhibit the maturation of DCs^26^ and leading to defects in T-cell priming and decreased T cell immunity response^27^. Reduced of DCs in stroma may be a response under the stimulation of cigarette smoke. However, we supposed the functional development of DCs was impaired during cigarette smoking induced carcinogenesis given that increased number but suppressed maturation of DCs 28 was observed in airway, lung parenchyma, and lymph nodes of mice after long-term cigarette smoke exposure^29^-^32^. In addition, less activated CTLs infiltrations in both intraepithelial and stromal region in the smokers' tumor specimens (Figure 2H), implying a suppressed immune response in TME during cigarette smoking exposure, also support the hypothesis. Defects of DCs maturation could also influence the Treg induction, which was presented higher infiltration in intraepithelial regions of cigarette smokers in Figure 2E, because immature DCs were converted into tolerogenic DC which secreted higher levels of TGF-β1, a cytokine triggering the conversion of naïve T cells into Treg cells^33^, ^34^. In ESCC, Treg infiltration was adversely correlated with survival of the patients^35^, ^36^ while granzyme B^+^ CTLs mediated the apoptotic death of tumor cells^37^. It would be very interesting to investigate the significance of the effects of cigarette smoke on DCs development and the regulation network among difference immune subset populations in ESCC microenvironment in an *in vivo* study.

Elder people always have higher risk for cancer, while elder smokers have longer smoking duration. Mutation accumulation was one of the explanations. And in our study, we found a positive correlation between age and Treg in intraepithelial region, as well as negatively with NK cells in stromal regions, in non-smoking patients(Table S3). However, age presented more complex connection with NK and TAM in smokers. After age-adjusting, only TAM infiltrating in stromal regions was associated with smoking year. Intriguingly, TAM did not differ between smokers and non-smokers either interepithelial or stromal regions, while other changed immune infiltrating cells (aCTL e.g.) between smokers and non-smokers showed no such dose-dependent relation. A similar study of Meredith etc.^38^, which reported a lower level of serum stem cell factor (SCF) and soluble interleukin 6 receptor (sIL-6R) in current smokers but increased association with smoking duration, supported our results. It is possible that the trigger of cigarette smoke exposure in carcinogenesis and effect of dose-response relationship of immune response involved in different regulation mechanisms, just like the diverse pathways during tumor malignant transformation, invasion, and migration. In addition, the synergic effect of different constituents of cigarette smoke and smoking regimens may also contribute to the immune infiltrating levels since it was a real exposure status of our study and it is possible to collect all the variables. In fact, many studies involved cigarette smoking did not reported the association of intensity of active cigarette smoke and their variables^10^, ^39^, ^40^.

PD-L1 expression was analyzed because several studies revealed that smoking status predicts different prognosis in immunotherapy^19^, ^41^. In association between PD-L1 and ESCC, majority of studies revealed patients of PD-L1 overexpression got poor clinical outcomes^42^-^44^ while some of them demonstrated that higher PD-L1 expressed level was related to favorable prognosis^45^-^47^. Our results may partly explain these inconsistent reports, since increased PD-L1^+^ cell in intraepithelial regions in nonsmokers indicated poor survival but not in smokers(Figure 4 and 5). Though limited specimens in our study, the difference impacts of PD-L^+^ level on smokers and non-smokers were obvious. A study of larger sample size in NSCLC, in which no significant increase of PD-L1^+^ cells was observed in smokers, either^48^, validated our observations. Few research explored the role of cigarette smoke in ESCC tumor environment, especially PD-L1 expression. However, the difference may be attributed to higher gene mutation frequency and immune environment difference in smokers 49-51. Therefore, the finding should be confirmed in further investigations with sufficient sample size and biomarkers while smoking status should be taken to consideration when evaluating the response to immune checkpoint inhibitors in ESCC as well as NSCLC.

Finally, our study has several notable limitations. First, small sample size of non-smokers would increase the likelihood of false negative, since we had to exclude the females that none of whom smoked at diagnosis to avoid the bias of gender. Second, genetic changes, tumor mutation burden e.g., should be taken consideration for a better explanation of the effects of cigarette smoking. Lastly, further studies, *in vitro* experiments, could be performed in future to explore the role in cigarette smoke-induced tumor environment changes.

In summary, our study revealed that cigarette smoking has a significantly immunosuppressive effect in altering tumor microenvironment, especially for T cell-mediated antitumor immunity. A complex characteristics and immune cells need further investigation. We highlighted the potential role of smoking status in association of PD-L ^+^ expression of prognosis.

## Supplementary Material

Supplementary figures and tables.

## Figures and Tables

**Table 1:** Multiple Cox regression OS of clinical characteristic in smokers and non-smokers of ESCC(n=117)

Variables	Total (n=117)		Non-smoker (n=34)		Current smoker (n=83)	
	HR(95%CI)	*P*	HR(95%CI)	*P*	HR(95%CI)	*P*
Age						
≤ 58 yrs†	1		1		1	
> 58 yrs	1.89(1.09, 3.29)	0.023^*^	3.36(1.05, 10.69)	0.041^*^	1.92(0.97, 3.77)	0.060
Tumor size						
≤ 3.5 cm	1		1		1	
> 3.5 cm	1.04(0.59, 1.82)	0.889	2.53(0.52, 12.25)	0.250	0.80(0.41, 1.57)	0.518
T stage						
T1-2	1		1		1	
T3	1.57(0.80, 3.07)	0.184	0.60(0.17, 2.09)	0.419	2.14(0.82, 5.6)	0.119
N stage						
N0	1		1		1	
N1-3	3.12(1.74, 5.60)	<0.001**	2.83(0.86, 9.29)	0.086	3.34(1.63, 6.83)	<0.001**
G stage						
W/D‡	1		1		1	
M/D	0.76(0.32, 1.78)	0.529	0.75(0.21, 2.66)	0.651	1.04(0.30, 3.58)	0.947
P/D	0.90(0.47, 1.73)	0.752	0.48(0.12, 1.92)	0.301	1.59(0.59, 4.28)	0.355
Smoking status						
Non-smoker	1		-		-	
Current smoker	1.05(0.78, 1.42)	0.730	-	-	-	-

† Median age, ^*^ *P*<0.05, ^**^ *P*<0.05. ‡ W/D, well-differentiated; M/D, moderately differentiated; P/D, poorly differentiated.

## Abbreviations

EAC: esophageal adenocarcinoma
ESCC: Esophageal Squamous Cell Carcinoma
PAHs: polycyclic aromatic hydrocarbons
AhR: aryl hydrocarbon receptor
nAChR: nicotinic acetylcholine receptor
OS: overall survival
DFS: disease-free survival
AJCC: American Joint Committee on Cancer
mflHC: multiplex fluorescent immunohistochemistry
FFPE: formalin-fixed paraffin-embedded
ECs: epithelial cells
DCs: dendritic cells
Tmems: memory T cells
NK: natural killer cells
TAMs: tumor-associated macrophages
Tregs: regulatory T cells
CTLs: cytotoxic T lymphocytes
aCTLs: activate cytotoxic T lymphocytes
PD-L1: programmed death ligand 1

## References

[1] Bray F, Ferlay J, Soerjomataram I, Siegel RL, Torre LA, Jemal A (2018). Global cancer statistics 2018: GLOBOCAN estimates of incidence and mortality worldwide for 36 cancers in 185 countries. CA Cancer J Clin.

[2] Chen W, Zheng R, Baade PD, Zhang S, Zeng H, Bray F (2016). Cancer statistics in China, 2015. CA Cancer J Clin.

[3] Wang QL, Xie SH, Li WT, Lagergren J (2017). Smoking Cessation and Risk of Esophageal Cancer by Histological Type: Systematic Review and Meta-analysis. J Natl Cancer Inst.

[4] Wang M, Luo X, Xu S, Liu W, Ding F, Zhang X (2019). Trends in smoking prevalence and implication for chronic diseases in China: serial national cross-sectional surveys from 2003 to 2013. Lancet Respir Med.

[5] Prabhu A, Obi KO, Rubenstein JH (2013). Systematic review with meta-analysis: race-specific effects of alcohol and tobacco on the risk of oesophageal squamous cell carcinoma. Aliment Pharmacol Ther.

[6] Cook MB, Kamangar F, Whiteman DC, Freedman ND, Gammon MD, Bernstein L (2010). Cigarette smoking and adenocarcinomas of the esophagus and esophagogastric junction: a pooled analysis from the international BEACON consortium. J Natl Cancer Inst.

[7] Hecht SS (2006). Cigarette smoking: cancer risks, carcinogens, and mechanisms. Langenbecks Arch Surg.

[8] Zong D, Liu X, Li J, Ouyang R, Chen P (2019). The role of cigarette smoke-induced epigenetic alterations in inflammation. Epigenetics Chromatin.

[9] Li X, Li J, Wu P, Zhou L, Lu B, Ying K (2018). Smoker and non-smoker lung adenocarcinoma is characterized by distinct tumor immune microenvironments. Oncoimmunology.

[10] Li J, Li H, Zhang C, Zhang C, Wang H (2020). Integrative analysis of genomic alteration, immune cells infiltration and prognosis of lung squamous cell carcinoma (LUSC) to identify smoking-related biomarkers. Int Immunopharmacol.

[11] Seiler CL, Song JUM, Kotandeniya D, Chen J, Kono TJY, Han Q (2020). Inhalation exposure to cigarette smoke and inflammatory agents induces epigenetic changes in the lung. Sci Rep.

[12] Hamada T, Nowak JA, Masugi Y, Drew DA, Song M, Cao Y (2019). Smoking and Risk of Colorectal Cancer Sub-Classified by Tumor-Infiltrating T Cells. J Natl Cancer Inst.

[13] Hanahan D, Weinberg RA (2011). Hallmarks of cancer: the next generation. Cell.

[14] Lagergren J, Smyth E, Cunningham D, Lagergren P (2017). Oesophageal cancer. Lancet.

[15] Zheng Y, Chen Z, Han Y, Han L, Zou X, Zhou B (2020). Immune suppressive landscape in the human esophageal squamous cell carcinoma microenvironment. Nat Commun.

[16] Yao J, Cui Q, Fan W, Ma Y, Chen Y, Liu T (2020). Single-cell transcriptomic analysis in a mouse model deciphers cell transition states in the multistep development of esophageal cancer. Nat Commun.

[17] Shah MA, Kojima T, Hochhauser D, Enzinger P, Raimbourg J, Hollebecque A (2019). Efficacy and Safety of Pembrolizumab for Heavily Pretreated Patients With Advanced, Metastatic Adenocarcinoma or Squamous Cell Carcinoma of the Esophagus: The Phase 2 KEYNOTE-180 Study. JAMA Oncol.

[18] Pan C, Wang Y, Liu Q, Hu Y, Fu J, Xie X (2021). Phenotypic profiling and prognostic significance of immune infiltrates in esophageal squamous cell carcinoma. Oncoimmunology.

[19] Wang GZ, Zhang L, Zhao XC, Gao SH, Qu LW, Yu H (2019). The Aryl hydrocarbon receptor mediates tobacco-induced PD-L1 expression and is associated with response to immunotherapy. Nat Commun.

[20] Wang X, Ricciuti B, Alessi JV, Nguyen T, Awad MM, Lin X (2021). Smoking History as a Potential Predictor of Immune Checkpoint Inhibitor Efficacy in Metastatic Non-Small Cell Lung Cancer. J Natl Cancer Inst.

[21] Qiu F, Liang CL, Liu H, Zeng YQ, Hou S, Huang S (2017). Impacts of cigarette smoking on immune responsiveness: Up and down or upside down?. Oncotarget.

[22] Stack EC, Wang C, Roman KA, Hoyt CC (2014). Multiplexed immunohistochemistry, imaging, and quantitation: a review, with an assessment of Tyramide signal amplification, multispectral imaging and multiplex analysis. Methods.

[23] McGinnis LM, Ibarra-Lopez V, Rost S, Ziai J (2021). Clinical and research applications of multiplexed immunohistochemistry and in situ hybridization. J Pathol.

[24] Fang P, Li X, Dai J, Cole L, Camacho JA, Zhang Y (2018). Immune cell subset differentiation and tissue inflammation. J Hematol Oncol.

[25] Goncalves RB, Coletta RD, Silverio KG, Benevides L, Casati MZ, da Silva JS (2011). Impact of smoking on inflammation: overview of molecular mechanisms. Inflamm Res.

[26] Ning Y, Shen K, Wu Q, Sun X, Bai Y, Xie Y (2018). Tumor exosomes block dendritic cells maturation to decrease the T cell immune response. Immunol Lett.

[27] Marciscano AE, Anandasabapathy N The role of dendritic cells in cancer and anti-tumor immunity. Semin Immunol. 2021: 101481.

[28] Givi ME, Folkerts G, Wagenaar GT, Redegeld FA, Mortaz E (2015). Cigarette smoke differentially modulates dendritic cell maturation and function in time. Respir Res.

[29] D'Hulst A I, Vermaelen KY, Brusselle GG, Joos GF, Pauwels RA (2005). Time course of cigarette smoke-induced pulmonary inflammation in mice. Eur Respir J.

[30] Botelho FM, Nikota JK, Bauer CM, Morissette MC, Iwakura Y, Kolbeck R (2012). Cigarette smoke-induced accumulation of lung dendritic cells is interleukin-1alpha-dependent in mice. Respir Res.

[31] Robbins CS, Franco F, Mouded M, Cernadas M, Shapiro SD (2008). Cigarette smoke exposure impairs dendritic cell maturation and T cell proliferation in thoracic lymph nodes of mice. J Immunol.

[32] Danov O, Wolff M, Bartel S, Bohlen S, Obernolte H, Wronski S (2020). Cigarette Smoke Affects Dendritic Cell Populations, Epithelial Barrier Function, and the Immune Response to Viral Infection With H1N1. Front Med (Lausanne).

[33] Kushwah R, Wu J, Oliver JR, Jiang G, Zhang J, Siminovitch KA (2010). Uptake of apoptotic DC converts immature DC into tolerogenic DC that induce differentiation of Foxp3+ Treg. Eur J Immunol.

[34] Feuerer M, Hill JA, Mathis D, Benoist C (2009). Foxp3+ regulatory T cells: differentiation, specification, subphenotypes. Nat Immunol.

[35] Raghavan S, Quiding-Jarbrink M (2011). Regulatory T cells in gastrointestinal tumors. Expert Rev Gastroenterol Hepatol.

[36] Vacchelli E, Semeraro M, Adam J, Dartigues P, Zitvogel L, Kroemer G (2016). Immunosurveillance in esophageal carcinoma: The decisive impact of regulatory T cells. Oncoimmunology.

[37] Rousalova I, Krepela E (2010). Granzyme B-induced apoptosis in cancer cells and its regulation (review). Int J Oncol.

[38] Shiels MS, Katki HA, Freedman ND, Purdue MP, Wentzensen N, Trabert B (2014). Cigarette smoking and variations in systemic immune and inflammation markers. J Natl Cancer Inst.

[39] de la Iglesia JV, Slebos RJC, Martin-Gomez L, Wang X, Teer JK, Tan AC (2020). Effects of Tobacco Smoking on the Tumor Immune Microenvironment in Head and Neck Squamous Cell Carcinoma. Clin Cancer Res.

[40] Piaggeschi G, Rolla S, Rossi N, Brusa D, Naccarati A, Couvreur S (2021). Immune Trait Shifts in Association With Tobacco Smoking: A Study in Healthy Women. Front Immunol.

[41] Wei Y, Li Y, Du Q, Peng X, Jin J, Guo H (2020). Effects of Clinicopathological Characteristics on the Survival of Patients Treated with PD-1/PD-L1 Inhibitor Monotherapy or Combination Therapy for Advanced Cancer: A Systemic Review and Meta-Analysis. J Immunol Res.

[42] Yagi T, Baba Y, Ishimoto T, Iwatsuki M, Miyamoto Y, Yoshida N (2019). PD-L1 Expression, Tumor-infiltrating Lymphocytes, and Clinical Outcome in Patients With Surgically Resected Esophageal Cancer. Ann Surg.

[43] Shi F, Xiao S, Miller KB, Zhao Y, Li Y, Gao Y (2020). Interactive Effects of PD-L1 Expression in Tumor and Immune Cells on Prognosis of Esophageal Squamous Cell Carcinoma: A One-Center Retrospective Cohort Study. Onco Targets Ther.

[44] Liang MQ, Yu FQ, Chen C (2020). C-Myc regulates PD-L1 expression in esophageal squamous cell carcinoma. Am J Transl Res.

[45] Wakita A, Motoyama S, Nanjo H, Sato Y, Yoshino K, Sasaki T (2017). PD-L1 Expression Is a Prognostic Factor in Patients with Thoracic Esophageal Cancer Treated Without Adjuvant Chemotherapy. Anticancer Res.

[46] Hatogai K, Fujii S, Kitano S, Kojima T, Daiko H, Yoshino T (2020). Relationship between the immune microenvironment of different locations in a primary tumour and clinical outcomes of oesophageal squamous cell carcinoma. Br J Cancer.

[47] Chen K, Cheng G, Zhang F, Zhang N, Li D, Jin J (2016). Prognostic significance of programmed death-1 and programmed death-ligand 1 expression in patients with esophageal squamous cell carcinoma. Oncotarget.

[48] Calles A, Liao X, Sholl LM, Rodig SJ, Freeman GJ, Butaney M (2015). Expression of PD-1 and Its Ligands, PD-L1 and PD-L2, in Smokers and Never Smokers with KRAS-Mutant Lung Cancer. J Thorac Oncol.

[49] Yokoyama A, Kakiuchi N, Yoshizato T, Nannya Y, Suzuki H, Takeuchi Y (2019). Age-related remodelling of oesophageal epithelia by mutated cancer drivers. Nature.

[50] Zang YS, Dai C, Xu X, Cai X, Wang G, Wei J (2019). Comprehensive analysis of potential immunotherapy genomic biomarkers in 1000 Chinese patients with cancer. Cancer Med.

[51] Yang X, Shi Y, Li M, Lu T, Xi J, Lin Z (2019). Identification and validation of an immune cell infiltrating score predicting survival in patients with lung adenocarcinoma. J Transl Med.

