# Supplementary material for: Impacts of Cigarette Smoking on the Tumor Immune Microenvironment in Esophageal Squamous Cell Carcinoma
Source: J Cancer. 2022 Jan 1;13(2):413–25. doi: 10.7150/jca.65400 (PMC8771511; doi:10.7150/jca.65400)
Supplement: Supplementary file 1 — Supplementary figures and tables. [file jcav13p0413s1.pdf]

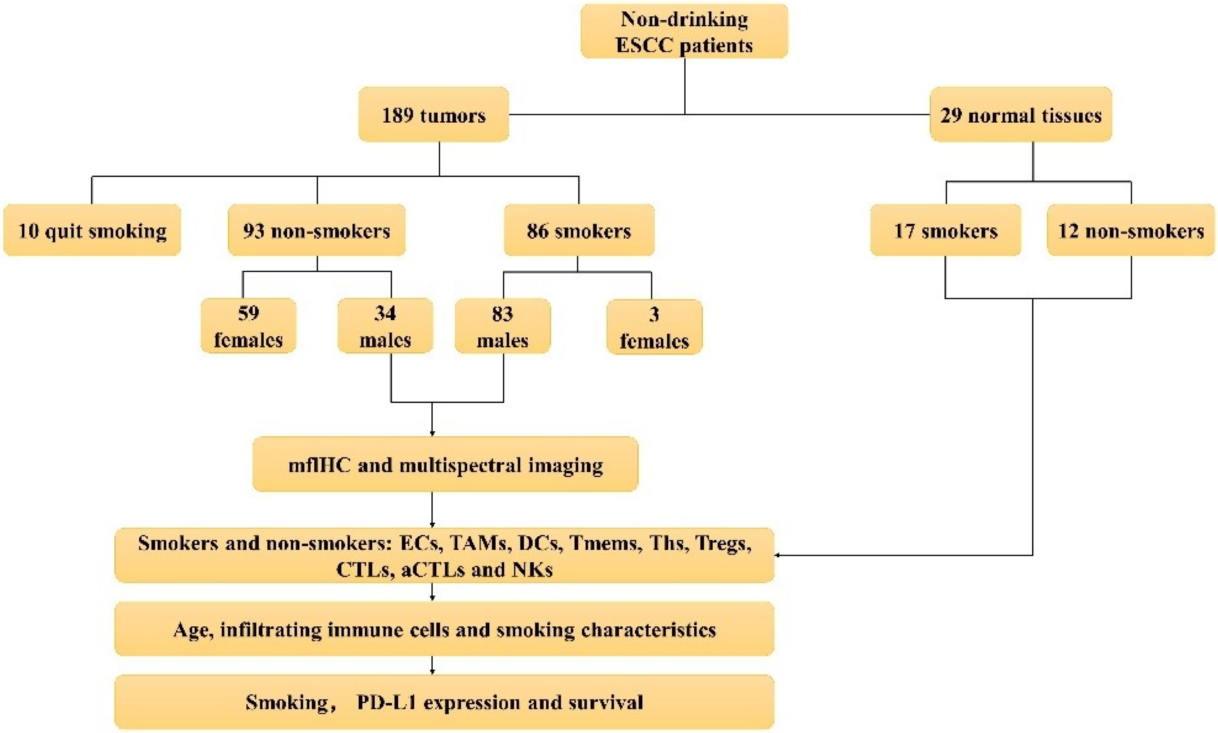

Figure S1. Overview flowchart of the study.

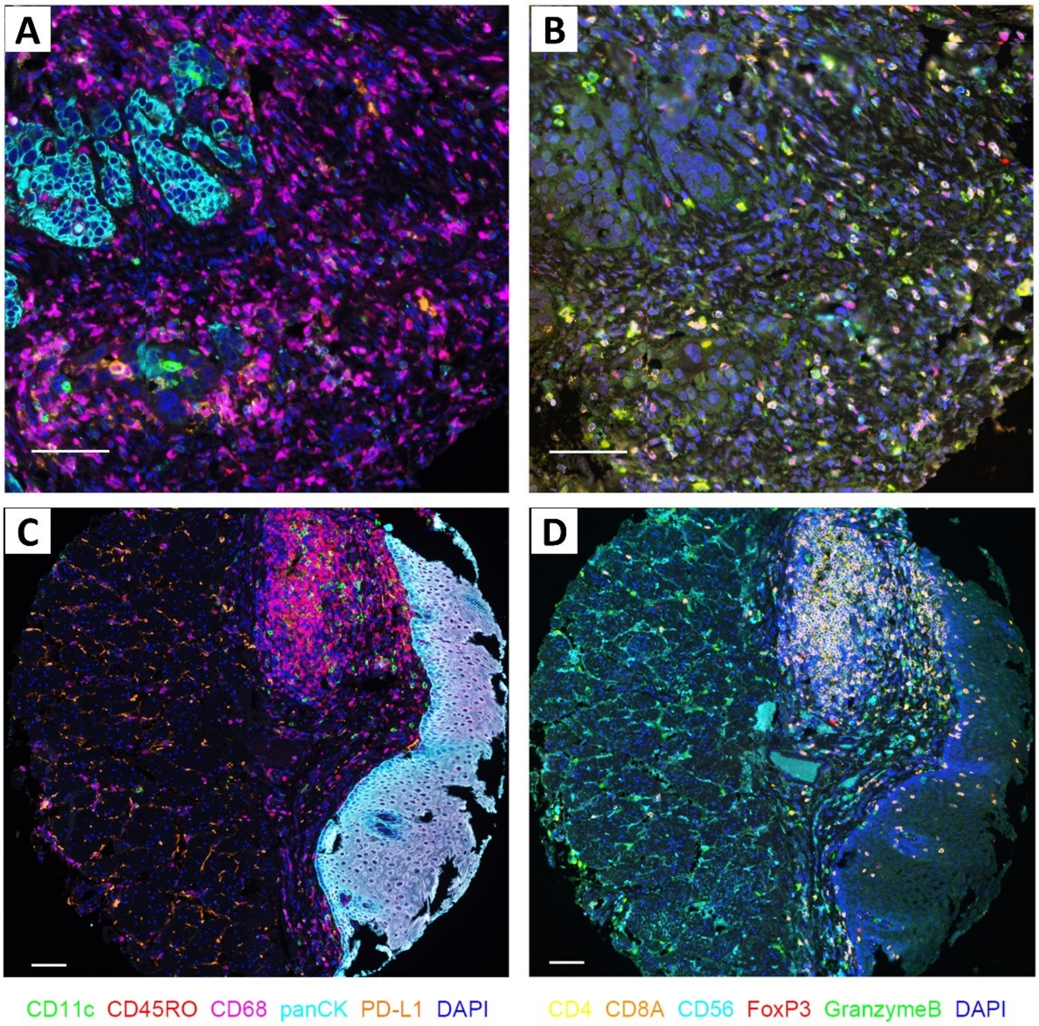

Figure S2. The representative images displaying different immune subpopulation in ESCC tumor specimens after multispectral merged imaging for panel A (A and C) and panel B (B and D).

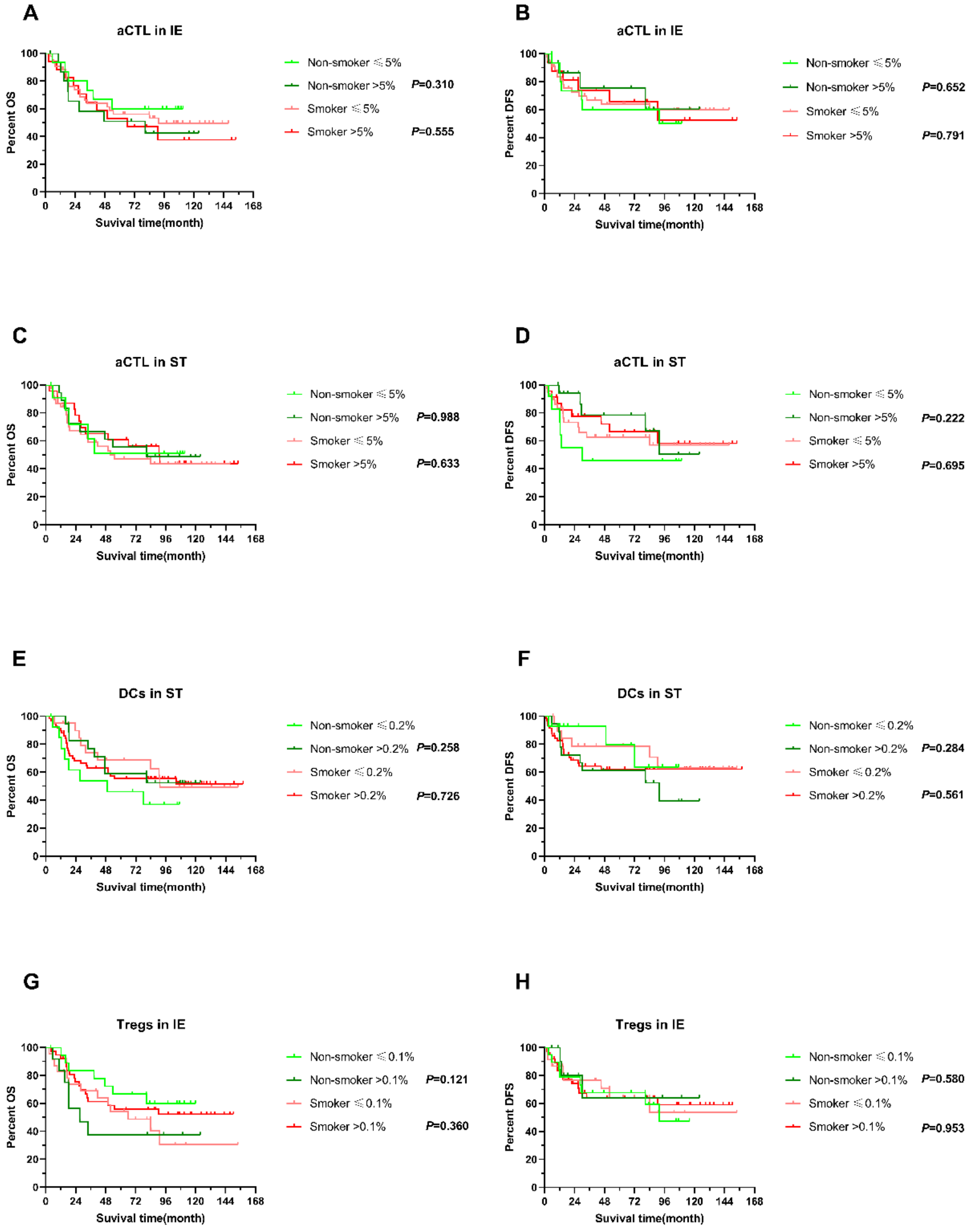

Figure S3. OS of stratified immune cell infiltration in tumor specimens between smoking and non-smoking ESCC patients.

Table S1 Clinical characteristics of patients with normal specimens

Variables	Total	Smoking status, n(%) Non-smoker (n=17)	Current smoker (n=12)	*P*
**Gender**				**0.001**[**]
**Male**	19	7 (36.8)	12 (63.2)	
**Female**	10	10 (100.0)	0 (0.0)	
**Age**				0.999
**≤ 58 yrs**	9	5 (55.6)	4 (44.4)	
**> 58yrs**	20	12 (60.0)	8 (40.0)	
**Tumor size**				0.408
**≤ 3.5cm**	21	11 (52.4)	10 (47.6)	
**> 3.5cm**	8	6 (75.0)	2 (25.0)	
**T stage**				0.273
**T1-2**	11	8 (72.7)	3 (27.3)	
**T3**	18	9 (50.0)	9 (50.0)	
**N stage**				0.999
**N0**	15	9 (60.0)	6 (40.0)	
**N1-3**	14	8 (57.1)	6 (42.9)	
**TNM stage**				0.471
**I-II**	17	11 (64.7)	6 (35.3)	
**III-IV**	12	6 (50.0)	6 (50.0)	
**G stage**				0.488
**W/D**[a]	3	1 (33.3)	2 (66.7)	
**M/D**	15	8 (53.3)	7 (46.7)	
**P/D**	11	8 (72.7)	3 (27.3)	

[a] W/D, well-differentiated; M/D, moderately differentiated; P/D, poorly differentiated.

[**] $P<0.01$.

Table S2 Clinical characteristics of patient with cancer specimens

Variables	Total	Smoking status, n(%)		*P*
		Non-smoker (n=34)	Current smoker (n=83)	
**Age**				0.226
**≤ 58 yrs†**	61	15(24.6)	46(75.4)	
**> 58yrs**	56	19(33.9)	37(66.1)	
**Tumor size**				0.489
**≤ 3.5cm**	54	14(25.9)	40(74.1)	
**> 3.5cm**	63	20(31.7)	43(68.3)	
**T stage**				0.092
**T1-2**	29	12(41.4)	17(58.6)	
**T3**	88	22(25.0)	66(75.0)	
**N stage**				
**N0**	56	14(25.0)	42(75.0)	0.354
**N1-3**	61	20(32.8)	41(67.2)	
**TNM stage**				0.869
**I-II**	64	19(29.7)	45(70.3)	
**III-IV**	53	15(28.3)	38(71.7)	
**G stage**				0.087
**W/D ‡**	30	8(26.7)	22(73.3)	
**M/D**	61	14(23.0)	47(77.0)	
**P/D**	26	12(46.2)	14(53.8)	

† Median age.

‡ W/D, well-differentiated; M/D, moderately differentiated; P/D, poorly differentiated.

Table S3 Correlation between age and immune infiltration in patient of ESCC

Variables†	Non-smokers			Smokers		
	n	r_s‡	*P*	n	r_s	*P*
**IE TAM**	32	-0.01	0.970	79	0.33	**0.003****
**ST TAM**	32	-0.10	0.600	78	0.36	**0.001****
**IE DC**	32	-0.14	0.458	79	0.10	0.372
**ST DC**	32	-0.27	0.135	78	0.10	0.370
**IE Tmem**	32	0.09	0.632	79	-0.10	0.357
**ST Tmem**	32	0.06	0.746	78	-0.19	0.089
**IE Treg**	31	0.39	**0.032***	60	-0.16	0.217
**ST Treg**	30	0.07	0.711	61	0.00	0.988
**IE Th**	31	-0.29	0.115	60	-0.03	0.828
**ST Th_s**	30	-0.06	0.738	61	0.07	0.617
**IE CTL**	31	0.31	0.087	60	0.09	0.477
**ST CTL**	30	0.21	0.269	61	0.00	0.990
**IE aCTL**	31	0.28	0.129	60	0.09	0.515
**ST aCTL**	30	0.06	0.736	61	0.01	0.987
**IE aCTL/CTL**	31	-0.22	0.230	60	0.02	0.891
**ST aCTL/CTL**	30	-0.30	0.111	61	-0.03	0.818
**IE NK**	31	-0.37	**0.043***	60	0.25	0.058
**ST NK**	30	-0.28	0.139	61	0.39	**0.002****

† IE, intraepithelial. ST, stromal. ‡r_s, Spearman correlation coefficient.

* $P<0.05$, ** $P<0.01$

Table S4 Correlation between age, smoking characteristics, and infiltrated cell in tissues of smoking ESCC patients

Infiltrating cell†	n	Age		Pack-years of smoking		Age-adjusted pack-years of smoking		Smoking year		Age-adjusted smoking year	
		r_s‡	***P***	r_s	***P***	r_s	***P***	r_s	***P***	r_s	***P***
**IE TAM**	79	0.33	**0.003****	0.11	0.347	0.04	0.733	0.26	**0.020***	0.12	0.312
**ST TAM**	78	0.36	**0.001****	0.25	**0.029***	0.19	0.105	0.47	**<0.001****	0.35	**0.002****
**IE DC**	79	0.10	0.372	0.08	0.472	0.06	0.590	-0.02	0.855	-0.09	0.461
**ST DC**	78	0.10	0.37	0.15	0.188	0.13	0.252	0.09	0.433	0.04	0.707
**IE Tmem**	79	-0.11	0.357	-0.13	0.27	-0.11	0.355	-0.18	0.104	-0.15	0.181
**ST Tmem**	78	-0.19	0.089	0.07	0.566	0.11	0.328	-0.06	0.595	0.05	0.696
**IE Treg**	60	-0.16	0.217	-0.25	0.056	-0.21	0.118	-0.23	0.081	-0.16	0.214
**ST Treg**	61	0.00	0.988	-0.12	0.358	-0.13	0.338	-0.06	0.629	-0.08	0.563
**IE Th**	60	-0.03	0.828	-0.01	0.972	0.01	0.963	0.03	0.802	0.06	0.633
**ST Th**	61	0.07	0.617	0.05	0.704	0.03	0.808	0.13	0.303	0.12	0.371
**IE CTL**	60	0.09	0.477	0.15	0.26	0.12	0.354	0.10	0.444	0.06	0.676
**ST CTL**	61	0.00	0.99	0.08	0.56	0.08	0.543	0.08	0.561	0.09	0.489
**IE aCTL**	60	0.09	0.515	0.15	0.263	0.13	0.347	0.16	0.234	0.13	0.321
**ST aCTL**	61	0.00	0.987	0.01	0.953	0.01	0.948	0.01	0.971	0.01	0.958
**IE aCTL/CTL**	60	0.02	0.891	0.06	0.627	0.06	0.642	0.14	0.294	0.16	0.227
**ST aCTL/CTL**	61	-0.03	0.818	-0.06	0.648	-0.05	0.687	-0.05	0.728	-0.04	0.792
**IE NK**	60	0.25	0.058	-0.04	0.737	-0.15	0.266	0.14	0.278	-0.01	0.943
**ST NK**	61	0.39	**0.002****	0.00	0.994	-0.13	0.317	0.18	0.168	-0.04	0.759

† IE, intraepithelial. ST, stromal. r_s, Spearman correlation coefficient

* P<0.05, ** P<0.01
